# Simabra Cedron

**Published:** 1855-01

**Authors:** 


**OBSERVATIONS ON SIMABA CEDRON. By S. S. Purple, M. D., N. Y.**

This is a pamphlet of sixteen pages, devoted to the discussion of the remedial powers of the above article in the control of intermittent fevers, together with a history and botanical description of the tree.

It recites cases in which cures have been effected when quinine had failed even. It grows in Central America, where it has been used with success in the cure of the bite of the most poisonous reptiles. Dr. Purple procured a package of the seeds and tested, in several cases, the value of this new antiperiodic in intermittents.—The result was highly satisfactory to him, and the facts thus gleaned he has embodied in this pamphlet, for the benefit of the profession. Dr. Magrath, it appears, has used it profitably in yellow fever, as well as intermittent, on the Isle of Jamaica. It is claimed that it produces less cephalic manifestations, and that its powers as an antiperiodic are equal, or nearly, to those of quinine.

The value of the discovery, in our opinion, will rest upon the abundance and cheapness of the article. We cannot hope to procure better antiperiodic or febrifuge properties in any thing than the bark or its preparations. But its cost is great, and any article which will make a good substitute and cost less, will prove a desideratum. We have had at different times *cornus florida*, *capsicum*, *arsenic*, *chlor sodium*, *&c.*, each and all proclaimed as being equal to the quinine, and yet, after all, when we look for certain results, we resort to bark or its salt, the quinine. We shall bide our time, and note the results, as they are developed by experiment and observation.

The pamphlet is well written, and the facts it records highly interesting.

